# Multiple Analyses of G-Protein Coupled Receptor (GPCR) Expression in the Development of Gefitinib-Resistance in Transforming Non-Small-Cell Lung Cancer

**DOI:** 10.1371/journal.pone.0044368

**Published:** 2012-10-29

**Authors:** Naoko Kuzumaki, Atsuo Suzuki, Michiko Narita, Takahiro Hosoya, Atsumi Nagasawa, Satoshi Imai, Kohei Yamamizu, Hiroshi Morita, Tsutomu Suzuki, Yohei Okada, Hirotaka James Okano, Jun K. Yamashita, Hideyuki Okano, Minoru Narita

**Affiliations:** 1 Department of Physiology, Keio University, School of Medicine, Tokyo, Japan; 2 Department of Pharamacology, Hoshi University School of Pharmacy and Pharmaceutical Sciences, Tokyo, Japan; 3 Biological Systems Control Team, Biomedicinal Information Research Center, Tokyo, Japan; 4 Laboratory of Stem Cell Differentiation, Stem Cell Research Center, Institute for Frontier Medical Sciences, Kyoto University, Kyoto, Japan; 5 Faculty of Pharmaceutical Sciences, Hoshi University School of Pharmacy and Pharmaceutical Sciences, Tokyo, Japan; 6 Department of Toxicology, Hoshi University School of Pharmacy and Pharmaceutical Sciences, Tokyo, Japan; National Taiwan University Hospital, Taiwan

## Abstract

There is increasing evidence that functional crosstalk between GPCRs and EGFR contributes to the progression of colon, lung, breast, ovarian, prostate and head and neck tumors. In this study, we performed multiple analyses of GPCR expression in a gefitinib-resistant non-small cell lung cancer (NSCLC) cell line, H1975, which harbors an L858R/T790M mutation. To determine the expression profile of mRNAs encoding 384 GPCRs in normal human lung fibroblast (NHLF) and H1975 cells, a GPCR-specific microarray analysis was performed. A heat-map of the microarray revealed considerable differences in the expression of GPCRs between NHLF and H1975 cells. From the GPCR expression list, we selected some GPCR agonists/antagonist to investigate whether the respective ligands could affect the growth of H1975 cells. Among them, treatment with either a selective antagonist of adenosine A2a receptors, which were highly expressed in H1975 cell and another gefitinib-resistant NSCLC cells, HCC827GR cells or “small interfering RNA” (siRNA) targeting adenosine A2a receptors produced a significant decrease in cell viability of both H1975 and HCC827GR cells. Among up-regulated GPCRs in H1975 cells, Gs-, Gi- and Gq-coupled GPCRs were expressed almost equally. Among down-regulated GPCRs, Gi-coupled GPCRs were dominantly expressed in H1975 cells. The present results suggest that multilayered crosstalk between GPCRs and EGFR may play an important role in orchestrating downstream signaling molecules that are implicated in the development of gefitinib-resistant NSCLC.

## Introduction

Non-small-cell lung cancer (NSCLC) is the leading cause of death from cancer. While, chemotherapy can slightly prolong survival in patients with advanced disease, It is also associated with clinically significant adverse effects [1]. Epidermal growth factor receptor (EGFR) is a major target of molecular anti-NSCLC therapy [2]. Gefitinib targets the ATP cleft in the tyrosine kinase EGFR, which is overexpressed in 40–80 percent of NSCLC and many other epithelial cancers [3]. EGFR signaling is triggered by the binding of growth factors, such as EGF, which results in either the dimerization of EGFR molecules or heterodimerization with related receptors, such as HER2. Autophosphorylation and transphosphorylation of EGFRs through their tyrosine kinase domains recruits downstream effectors and activates signals for proliferation and cell-survival [4]. Mutations have been identified in the EGFR gene in specimens from patients with NSCLC who respond to EGFR inhibitors [5]. These mutations consist of small deletions that affect amino acids 746 through 750 (delE746-A750) or point mutations (most often leucine replaced by arginine at codon 858 [L858R]) [6], [7], [8]. These mutations modify downstream signaling and antiapoptotic mechanisms, and thus mediate oncogenic effects [9]. Both of these mutations make the tumor more sensitive to compounds that inhibit EGFR, most likely by repositioning critical residues that surround the ATP-binding cleft of the tyrosine kinase domain of the receptor, which stabilizes interactions with both ATP and its competitive inhibitors [6], [7]. In our case, DNA sequencing of the EGFR gene in a tumor biopsy specimen at relapse showed a second point mutation that changed threonine to methionine at position 790 (T790M) of EGFR [5]. The efficacy of gefitinib is of limited duration, mainly due to drug resistance conferred by a second point mutation.

The activity of Akt, which is also known as protein kinase B (PKB), is stimulated by various growth factors, and this serine-threonine kinase plays evolutionarily conserved roles in many cellular functions, such as protein synthesis and cell growth [10], [11]. It has been reported that EGFR inhibitor changes strong, transient Akt phosphorylation to weak, sustained Akt phosphorylation. Due to the low-pass filter characteristics of the Akt pathway, this leads to the stronger phosphorylation of S6, which is a molecule downstream of Akt, than that in the absence of the inhibitor. Thus, EGFR inhibitor could act as a downstream activator of EGFR [12]. Taken together, these findings suggest that the process of gefitinib-resistance leads to the exacerbation of tumor cells.

A large body of evidence indicates that G protein-coupled receptors (GPCRs) play a crucial role in tumorigenesis, and are implicated in important steps in cancer progression from transformation, growth and survival to metastasis. Another important way that GPCRs contribute to tumorigenesis involves intensive crosstalk with a canonical pathway. There is considerable evidence that agonists of some GPCRs, through a process called transactivation, can activate growth factor receptor tyrosine kinases (RTKs) in the absence of added growth factor [13], [14], [15]. This is an important pathway that contributes to the growth-promoting activity of many GPCR ligands. On the other hand, recent findings have indicated that RTKs transduce signals through the use GPCR signaling molecules and RTK ligands themselves can transactivate GPCRs. To mediate tumor survival and proliferation, GPCRs may interact with EGFR downstream signaling pathways, including phosphatidylinositol 3-kinase (PI3K)/Akt, and Janus kinase/signal transducers and activators of transcription (Jak/Stat3) pathways. Indeed, the functional crosstalk between GPCRs and EGFR contributes to the progression of colon, lung, breast, ovarian, prostate and head and neck tumors [16], [17]. Thus, GPCR could be an excellent site for blocking tumorigenic signals, which would make GPCR-mediated functions promising therapeutic targets in drug development to achieve innovative intervention in NSCLC. In the present study, we performed multiple analyses of GPCR expression in a gefitinib-resistant NSCLC cell line, H1975.

## Materials and Methods

### Cell culture

The human non-small cell lung cancer cell (NSCLC) lines HCC827, NCI-H1975 (H1975; American Type Culture Collection Co., MD, USA) and HCC827GR were cultured in RPMI 1640 medium HEPES Modification (Sigma-Aldrich Co., MO, USA) with 10% fetal bovine serum (FBS; Invitrogen™ Life Technologies Co., CA, USA) and 1% penicillin-streptomycin (PS; Invitrogen™ Life Technologies Co.). Normal human lung fibroblasts (NHLF; Lonza Inc., NJ, USA) were cultured in fibroblast basal medium with insulin, rhFGF-B, GA-1000 and FBS (all from Takara Bio Inc., Tokyo, Japan). All cells were maintained under a humidified atmosphere of 5% CO_2_ at 37°C.

### Establishment of the gefitinib-resistant cell line, HCC827GR

HCC827 cells were exposed to 1 µM of gefitinib for 48 h in medium containing 10% fetal bovine serum. They were then washed and cultured in drug-free medium until surviving cells were 80% confluent. These cells were then re-exposed to increasing concentrations of gefitinib (from 1 to 5 µM). Cells were finally able to grow in 5 µM gefitinib were obtained 1.5 month after initial exposure. The established resistant cells were maintained in medium containing 1 µM of gefitinib. For all in vitro studies, resistant cells were cultured in drug-free medium for at least 1 week to eliminate gefitinib. Gefitinib-resistant cells are referred to as HCC827GR.

### Reagents

The reagents used in the present study were gefitinib (Toronto Research Chemicals Inc., Canada), [Arg^8^]-vasopressin acetate salt (Sigma Chemical Co., St. Louis, MO, USA), angiotensin II (Sigma Chemical Co.), 2-(methylthio) adenosine 5′-triphosphate tetrasodium salt hydrate (Sigma Chemical Co.), beraprost sodium (TORAY Industries Inc., Tokyo, Japan), a-methyl-5-(2-thienylmethoxy)-1H-Indole-3-ethanamine monohydrochloride (BW723C86; Sigma Chemical Co.), 2-*p*-(2-carboxyethyl) phenethylamino-5′-N-ethylcarboxamidoadenosine hydrochloride hydrate (CGS- 21680; Sigma Chemical Co.), 7-(2-phenylethyl)-5-amino-2- (2-furyl)- pyrazolo-[4,3-e]-1,2,4-triazolo [1,5-c] pyrimidine (SCH-58261; Sigma Chemical Co.).

### Cell viability assay

Cells viability was determined by the 3-(4,5-dimethylthiazol-2-yl)-2,5-diphenyl-tetrazolium bromide (MTT) assay. Twenty µL of MTT solution (5 mg/mL) was added to each well of the culture medium. After incubation for another 2 hr, the medium was removed, and 100 µL of DMSO were added to resolve formazan crystals. Optical density was measured made using a microplate reader at an absorption wavelength 600 nm. In each experiment, three replicates were prepared for each sample. The proportion of living cells was determined based on the difference in absorbance between samples and controls.

### GPCR TaqMan Array

A commercially available TaqMan Array Human GPCR card (Applied Biosystems Inc., Foster City, CA, USA) which allows us to quantify the expression of mRNAs that encode GPCRs from 50 subfamilies (343 receptors, not including the odorant, olfactory, gustatory and pheromone receptors), was used with RNA extracted from NHLF and H1975 cells. Total RNA, obtained from NHLF and H1975 cells, was extracted using the mirVana™ miRNA Isolation Kit (Applied Biosystems Inc.). For this assay, cDNA was prepared with a high-capacity RNA to cDNA kit (Applied Biosystems Inc.). Each port was loaded with cDNA (from 1.5 µg of RNA) and TaqMan Gene Expression Master Mix (Applied Biosystems Inc.) according to the manufacturer's instructions. The plate was analyzed using SDS2.3 and RQ Manager 1.2 software provided by Applied Biosystems. Cycle times were normalized aaato the housekeeping gene GAPDH. A comparative Ct approach was used to quantify the relative levels of mRNA using RQ 1.2 software. Relative expression levels were calculated as 2×10^5^. The results for each GPCR were examined, and if the sample had a calculated threshold below 0.1, it was considered to be undetectable. In these cases, for data processing purposes, the cycle number was set at 35.0.

### RNA preparation and semi-quantitative analysis by reverse transcription-polymerase chain reaction (RT-PCR)

Total RNA in NHLF, H1975, HCC827 and HCC827GR was extracted using the SV Total RNA Isolation system (Promega, Madison, WI) following the manufacturer's instructions. Purified total RNA was quantified spectrophotometrically at A260. To prepare first-strand cDNA, 1 µg of RNA was incubated in 100 µL of buffer containing 10 mM dithiothreitol, 2.5 mM MgCl_2_, dNTP mixture, 200 U of reverse transcriptase II (Invitrogen), and 0.1 mM oligo-dT12-18 (Invitrogen). Each gene was amplified in 50 µL of PCR solution containing 0.8 mM MgCl_2_, dNTP mixture, and DNA polymerase with synthesized primers of human A2a receptor (sense: GGCTGCCCCTACACATCATCAACT, antisense: TGGGCCAGGGGGTCATCT) and GPR87 (sense: 5′- CTCTAAAGGGGTAAGGGAGA-3′, antisense: 5′-TGGGTTCAGCATAGGTTATT-3′). Samples were heated at 95°C for 1 min, 55°C for 2 min, and 72°C for 3 min. The final incubation was at 72°C for 7 min. The mixture was run on 2% agarose gel electrophoresis with the indicated markers and primers for the internal standard glyceraldehyde-3-phosphate dehydrogenase. The agarose gel was stained with ethidium bromide and photographed with UV transillumination. The intensity of the bands was analyzed and semi-quantified by computer-assisted densitometry using ImageJ software.

### Sample preparation and Western blotting

Cells were solubilized with buffer containing 20 mM Tris-HCl (pH7.4), 0.3%(w/v) Triton, 3 mM MgCl_2_, 1 M sucrose, 5 mM α-ME, and 1/1,000 protease inhibitor for 15 min. Cell lysates were centrifuged at 2,350 g for 10 min at 4°C and the supernatant was retained as the lysate fraction for Western blotting. An aliquot of sample was diluted with an equal volume of 2× electrophoresis sample buffer (Protein Gel Loading Dye-2X, Amresco, Solon, OH) containing 2% sodium dodecyl sulfate (SDS) and 10% glycerol with 0.2 M dithiothreitol. Proteins (7 µL/lane) were separated by size on 4–20% SDS-polyacrylamide gradient gel and transferred to nitrocellulose membranes in Tris-glycine buffer containing 25 mM Tris and 192 mM glycine. For immunoblot detection, membranes were blocked in Tris-buffered saline (TBS) containing 1% nonfat milk (Bio-Rad Laboratories, Hercules, CA, USA) containing 0.1% Tween 20 (Research Biochemicals, Inc., MA, USA) for 1 h at room temperature with agitation. The membrane was incubated with primary antibody diluted in TBS [1∶1000 GPR87 (Abcam, Cambridge UK), 1∶200,000 glyceraldehyde-3-phosphate dehydrogenase (GAPDH; Chemicon International Inc., Temecula, CA, USA)] containing 1% nonfat dried milk with 0.1% Tween 20 overnight at 4°C. The membrane was washed in TBS containing 0.05% Tween 20 (TTBS), and then incubated for 2 h at room temperature with horseradish peroxidase-conjugated goat anti-rabbit IgG (Southern Biotechnology Associates, Inc., Birmingham, AL, USA) diluted 1∶10,000 in TBS containing 1% nonfat dried milk containing 0.1% Tween 20. After this incubation, the membranes were washed in TTBS. The antigen-antibody peroxidase complex was finally detected by enhanced chemiluminescence (Pierce, Rockford, IL, USA) according to the manufacturer's instructions and visualized by exposure to Amersham Hyperfilm (Amersham Life Sciences, Arlington Heights, IL, USA).

### “Small interfering RNA” (siRNA) transfection

H1975 and HCC827GR cells were transfected with commercially available “small interfering RNA” (siRNA) targeting adenosine A2a receptors (NIPPON EGT CO., Toyama, JAPAN) following reverse transfection method. The corresponding target mRNA sequence of adenosine A2a receptor for the siRNA was as follows: 5′- ACAGCAACCTGCAGAACGT-3′, (accession number: NM_000675.4). Briefly, adenosine A2a receptor gene-specific siRNA were diluted in Opti-MEM (Invitorogen) and mixed with RNAimax (Invitorogen) pre-diluted in Opti-MEM. After 20 min incubation at room temperature, the complexes were added to the 96-well. After that, cells (5×10^3^ cells) were seeded into that well. After 3 days culture, cell viability was detected by MTT assay.

### Statistical Analysis

All data are presented as the mean ± S.E.M. The statistical significance of differences between groups was assessed by one-way analysis of variance (ANOVA) followed by the Bonferroni/Dunn multiple comparison test. The statistical significance of differences between two groups was assessed with Student's *t*-test.

## Results

### Effect of the tyrosine kinase inhibitor gefitinib on the growth of non-small cell lung cancer (NSCLC) cells

**Figure 1:**
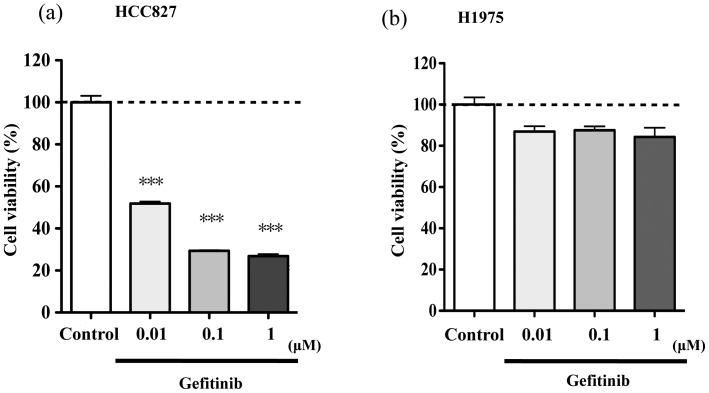
Effect of gefitinib on the growth of gefitinib-sensitive (HCC827) or resistant (H1975) NSCLC cell lines. HCC827 (a) or H1975 (b) cells were incubated for 2 days with gefitinib, and then cell viability was measured (***p<0.001 vs. non-treated group).

Addition of the tyrosine kinase inhibitor gefitinib (0.0001 µM–1 µM) to HCC827 cells, which have been classified as the gefitinib-sensitive NSCLC cell line [5], for 2 days produced a concentration-dependent decrease in tumor cell growth (**Figure 1a-i**, p<0.001 vs. non-treated group). In contrast, the addition of gefitinib (0.0001 µM–1 µM) for 2 days did not affect the growth of H1975 cells (**Figure 1a-ii**).

### Different expression of GPCRs between NHLF and H1975 cells

**Figure 2:**
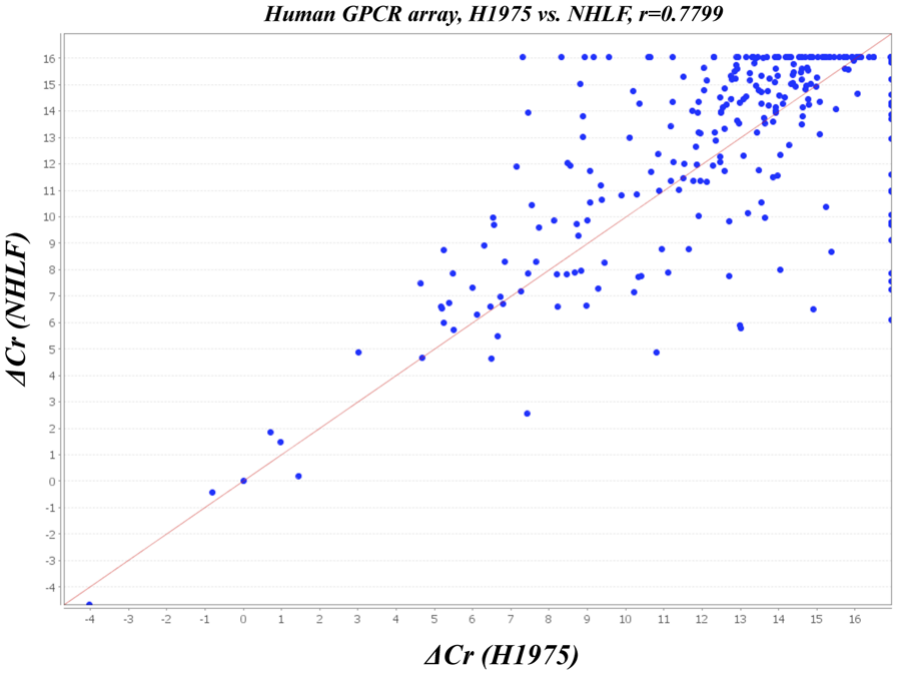
Pearson's correlation scatter plot of GPCR levels in NHLF and H1975 cells (R = 0.7799). Human GPCR expression was analyzed by microarrays.

To profile the expression of mRNAs encoding 384 GPCRs in NHLF and H1975 cells, a TaqMan GPCR-specific microarray analysis was performed (**Figures 2** **,** **3** **, Figures S1-1, S1-2, S1-3** for the list of GPCR genes in the array). The scatter plot showed that there were many more up-regulated GPCRs than down-regulated GPCRs in H1975 cells compared to NHLF. A heat-map of the microarray revealed considerable differences in the expression of GPCRs between NHLF and H1975 cells (**Figures 2** **,** **3**).

**Figure 3:**
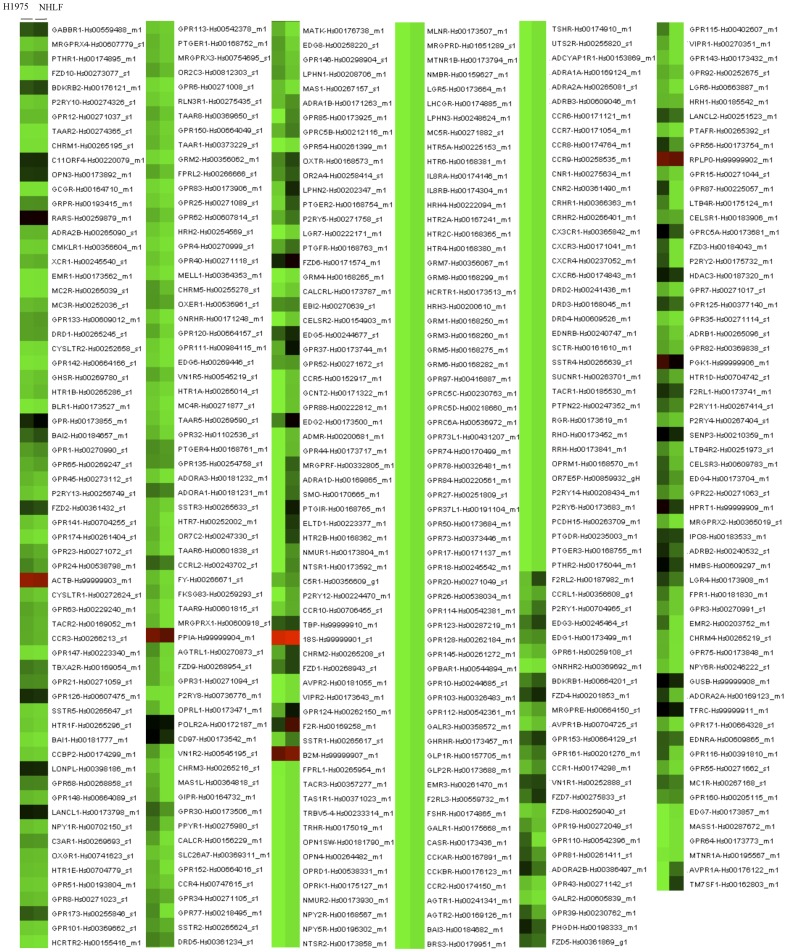
Heatmap of 384 human GPCR differentially expressed in H1975 cells compared to NHLF cells. Each GPCR is shown as a single bar based on their Ct values and color coding is shown below, with a gradient from green (negative and lowest Ct values) to red (positive and highest Ct values).

### Changes in the expression ratios of Gα subunits (Gs, Gi and Gq) in H1975 cells

The expression patterns of Gα subunits among up- or down-regulated GPCRs of H1975 cells compared to NHLF were assigned to the following groups: up-regulated Gs-coupled GPCR (**Table 1-a**), up-regulated Gi-coupled GPCR (**Table 1-b**), up-regulated Gq-coupled GPCR (**Table 1-c**), down-regulated Gs-coupled GPCR (**Table 2-a**), down-regulated Gi-coupled GPCR (**Table 2-b**) and down-regulated Gq-coupled GPCR (**Table 2-c**).

**Table 1:** G-coupled GPCR that were up-regulated in H1975 compared to NHLF (2≧fold change).

(a) Gs			
Symbol	Description	H1975/NHLF fold difference	Coupling
**GPR87**	G protein-coupled receptor 87	425.1676	Gs
**ADORA2A**	adenosine A2a receptor	90.3941	Gs
**GIPR**	gastric inhibitory polypeptide receptor	13.2001	Gs
**CALCR**	calcitonin receptor	13.1582	Gs
**ADORA2B**	adenosine A2b receptor	10.9389	Gs
**ADRB2**	adrenergic beta-2 receptor	8.849	Gs
**VIPR1**	vasoactive intestinal peptide receptor 1	8.7399	Gs
**LPHN1**	latrophilin 1	5.4757	Gs
**ADRB1**	adrenergic receptor beta 1	4.7792	Gs
**HTR7**	5-hydroxytryptamine (serotonin) receptor 7	4.3725	Gs
**P2RY11**	purinergic receptor P2Y G-protein coupled 11	3.5613	Gs,Gq
**MC1R**	melanocortin 1 receptor	2.3968	Gs
**MC4R**	melanocortin 4 receptor	2.2711	Gs
**DRD5**	dopamine receptor D5	2.0623	Gs

(b) Gi			
Symbol	Description	H1975/NHLF fold difference	Coupling
**PPYR1**	pancreatic polypeptide receptor 1	15.2541	Gi
**AGTRL1**	angiotensin II receptor-like 1	13.8131	Gi
**VN1R2**	vomeronasal 1 receptor 2	11.9937	Gi
**EDG4**	lysophosphatidic acid receptor 2	10.4805	Gi
**EDG8**	sphingosine-1-phosphate receptor 5	8.1024	Gi
**HTR1D**	5-hydroxytryptamine (serotonin) receptor 1D	7.4536	Gi
**OPRL1**	opiate receptor-like 1	6.594	Gi
**CHRM4**	cholinergic receptor, muscarinic 4	5.9843	Gi
**OR7C2**	olfactory receptor family 7 subfamily C member 2	5.467	Gi
**PTAFR**	platelet-activating factor receptor	2.8268	Gi,Gq
**F2RL1**	coagulation factor II (thrombin) receptor-like 1	2.7511	Gi,Gq
**GNRHR**	onadotropin-releasing hormone receptor	2.669	Gi,Gq
**ADORA3**	adenosine A3 receptor	2.5344	Gi
**CCR4**	chemokine (C-C motif) receptor 4	2.4829	Gi
**SSTR2**	somatostatin receptor 2	2.4233	Gi
**PTGER1**	prostaglandin E receptor 1	2.2708	Gi,Gq
**SSTR3**	omatostatin receptor 3	2.2642	
**EDG6**	lysophosphatidic acid G-protein-coupled receptor 6	2.0191	Gi

(c) Gq			
Symbol	Description	H1975/NHLF fold difference	Coupling
**P2RY2**	purinergic receptor P2Y G-protein coupled 2	209.5184	Gq
**FZD3**	frizzled homolog 3 (Drosophila)	137.3562	Gq
**EMR2**	egf-like module containing mucin-like hormone receptor-like 2	88.5791	Gq
**LTB4R**	leukotriene B4 receptor	41.6841	Gq
**FPR1**	formyl peptide receptor 1	23.6083	Gq
**LTB4R2**	leukotriene B4 receptor 2	8.6323	Gq
**MRGPRX2**	MAS-related GPR member X2	7.3656	Gq
**FZD9**	frizzled homolog 9 (Drosophila)	6.258	Gq
**CHRM5**	cholinergic receptor muscarinic 5	4.1532	Gq
**P2RY11**	purinergic receptor P2Y G-protein coupled 11	3.5613	Gq,Gs
**CHRM3**	cholinergic receptor muscarinic 3	3.2644	Gq
**PTAFR**	platelet-activating factor receptor	2.8268	Gq,Gi
**F2RL1**	coagulation factor II (thrombin) receptor-like 1	2.7511	Gq,Gi
**GNRHR**	onadotropin-releasing hormone receptor	2.669	Gq,Gi
**P2RY4**	pyrimidinergic receptor P2Y G-protein coupled, 4	2.5966	Gq
**FFAR2**	free fatty acid receptor 2	2.5666	Gq
**PTGER1**	prostaglandin E receptor 1	2.2708	Gq,Gi

**Table 2:** G-coupled GPCR that were down-regulated in H1975 compared to NHLF (0.5 ≦ fold change).

(a) Gs			
Symbol	Description	H1975/NHLF fold difference	Coupling
**PTGIR**	prostaglandin I2 (prostacyclin) receptor	0.0005	Gs
**PTGER2**	prostaglandin E receptor 2	0.0096	Gs
**EDG5**	sphingosine-1-phosphate receptor 2	0.1995	Gs
**LGR7**	relaxin/insulin-like family peptide receptor 1	0.3015	Gs
**AVPR2**	arginine vasopressin receptor 2	0.4646	Gs
**VIPR2**	vasoactive intestinal peptide receptor 2	0.4674	Gs

(b) Gi			
Symbol	Description	H1975/NHLF fold difference	Coupling
**F2RL2**	Coagulation factorII (thrombin) receptor-like 2	0.0152	Gi
**EDG2**	lysophosphatidic acid receptor 1	0.0162	Gi
**CHRM2**	cholinergic receptor muscarinic 2	0.0323	Gi
**F2R**	coagulation factor II (thrombin) receptor	0.0342	Gi
**SSTR1**	somatostatin receptor 1	0.0344	Gi
**P2RY12**	purinergic receptor P2Y G-protein coupled 12	0.0629	Gi
**OXTR**	oxytocin receptor	0.1066	Gi, Gq
**FZD1**	frizzled homolog 1 (Drosophila)	0.1587	Gi
**CCR5**	chemokine (C-C motif) receptor 5	0.1621	Gi
**EDG3**	sphingosine-1-phosphate receptor 3	0.1654	Gi
**CCR1**	chemokine (C-C motif) receptor 1	0.1975	Gi
**BDKRB1**	bradykinin receptor B1	0.2243	Gi, Gq
**CCR10**	chemokine (C-C motif) receptor 10	0.2559	Gi

(c) Gq			
Symbol	Description	H1975/NHLFfold difference	Coupling
**HTR2B**	5-hydroxytryptamine (serotonin) receptor 2B	0.0019	Gq
**MRGPRF**	MAS-related GPR member F	0.0044	Gq
**GPR37**	endothelin receptor type B-like	0.0066	Gq
**AVPR1A**	arginine vasopressin receptor 1A	0.0071	Gq
**ADRA1D**	adrenergic alpha 1D receptor	0.0086	Gq
**NTSR1**	neurotensin receptor 1	0.0159	Gq
**NMUR1**	neuromedin U receptor 1	0.0247	Gq
**P2RY1**	purinergic receptor P2Y G-protein coupled 1	0.0784	Gq
**OXTR**	oxytocin receptor	0.1066	Gq,Gi
**ADRA1B**	adrenergic alpha 1B receptor	0.1864	Gq
**BDKRB1**	bradykinin receptor B1	0.2243	Gq,Gi
**AVPR1B**	arginine vasopressin receptor 1B	0.3674	Gq

**Figure 4:**
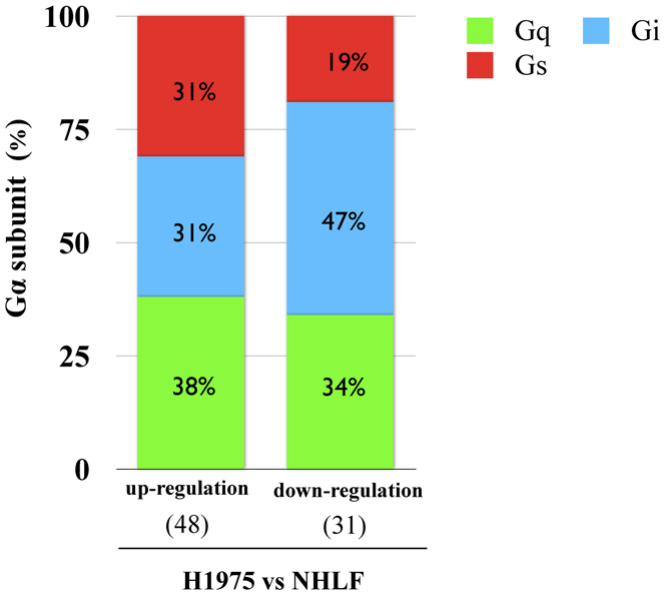
High levels of GPR87 expression in H1975 cells. (a) Upper: Representative RT-PCR for mRNAs of GPR87 and GAPDH, an internal standard, in each cell type. Lower: The intensity of the bands was determined semi-quantitatively using ImageJ. The values for GPR87 mRNA were normalized by the value for GAPDH mRNA. Data represent the mean with S.E.M. of 3 independent samples (***p<0.001 vs. NHLF). (b) Upper: Representative Western blots of GPR87 Lower: Representative Western blots of GPR87 in membranous fractions of H1975 cells. Each column represents the mean with S.E.M. of 3 independent samples (**p<0.001 vs. non-treated group).

The mRNA and its protein expressions of GRP87, which was the most up-regulated GPCR found in the GPCR array, were significantly up-regulated in H1975 cells compared to those in NHLF cells (Figure 4a: p<0.01 vs. NHLF, Figure 4b: p<0.001 vs. NHLF).

**Figure 5:**
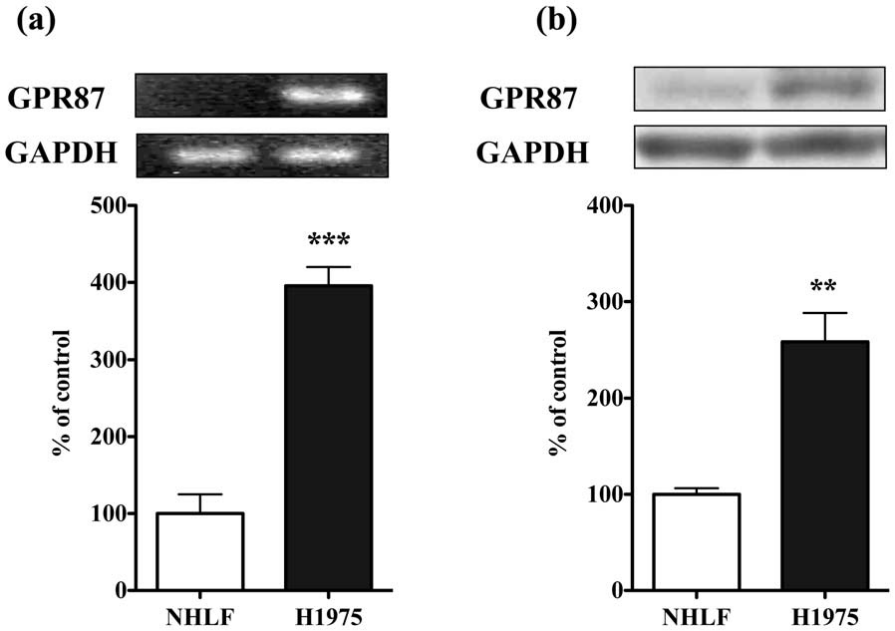
Expression ratio of Gα subunit (Gs, Gi and Gq) in H1975 cells. The percentage of expression pattern of Gα subunit (Gs: red, Gi: blue and Gq: green) in the up- or down-regulated GPCRs of H1975 cells compared to NHLF is shown.

Among up-regulated GPCRs in H1975 cells, Gs-, Gi- and Gq-coupled GPCRs were expressed almost equally. Among down-regulated GPCRs, Gi-coupled GPCRs were dominantly expressed in H1975 cells (**Figure 5**).

### Effects of a selective GPCR agonist or antagonist on the growth of gefitinib-resistant H1975 cells

**Figure 6:**
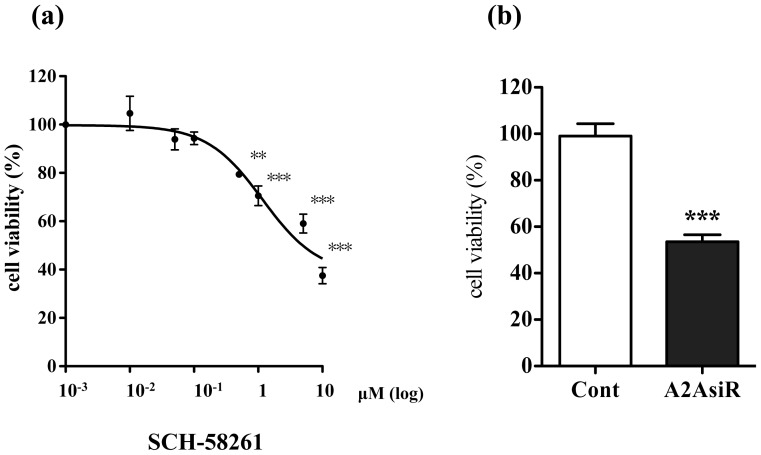
Effects of a selective antagonist of adenosine A2a receptors or siRNA targeting adenosine A2a receptors on the growth of H1975 cells. (a) H1975 cells were incubated for 7 days with the selective adenosine A2a receptor antagonist SCH-58261 (10 nM–10 µM), and then cell viability was measured (**p<0.01, ***p<0.001 vs. non-treated group). (b) Treatment with siRNA targeting adenosine A2a receptors to H1975 cells for 3 days significantly decreased cell viability of H1975 cells (**p<0.01 vs. non-treated group).

From the GPCR expression list, some commercially available GPCR agonist/antagonist were selected to investigate whether the respective ligands affected the growth of H1975 cells (**Figure S2**). An adenosine A2a receptor (CGS-21680), an angiotensin II receptor-like 1 agonist (angiotensin II) and a purinergic receptor P2Y G-protein coupled 2 agonist (2-(methylthio) adenosine 5′-triphosphate tetrasodium salt hydrate), which were all agonists of up-regulated GPCRs in the microarray list, had no effect on tumor cell growth. Similarly, cell growth was not affected by a prostaglandin I_2_ receptor (IP) agonist (beraprost sodium), a 5-hydroxytryptamine receptor 2B agonist (BW723C86) and an arginine vasopressin receptor 1A agonist ([Arg^8^]-vasopressin acetate salt), which were all classified as “down-regulated GPCRs”. In contrast, addition of the selective adenosine A2a receptor antagonist SCH-58261 (10 nM–10 µM) for 7 days (**Figure 6a**) produced a concentration-dependent decrease in H1975 cell growth (p<0.01, p<0.001 vs. non-treated group).

We next performed to transduce siRNA targeting adenosine A2a receptors into H1975 cells. Cells were harvested for detection of the adenosine A2a receptor mRNA level semi-quantitatively by RT-PCR at 72 h post-transfection. The adenosine A2a receptor expression in the presence of siRNA targeting adenosine A2a receptors was down-regulated by 90% compared to non-treatment (data not shown). Under these conditions, treatment with siRNA targeting adenosine A2a receptors produced a significant decrease in H1975 cell growth (**Figure 6b**: p<0.001 vs. non-treated group).

### Role of adenosine A2a receptors in the growth of gefitinib-resistant HCC827GR cells

**Figure 7:**
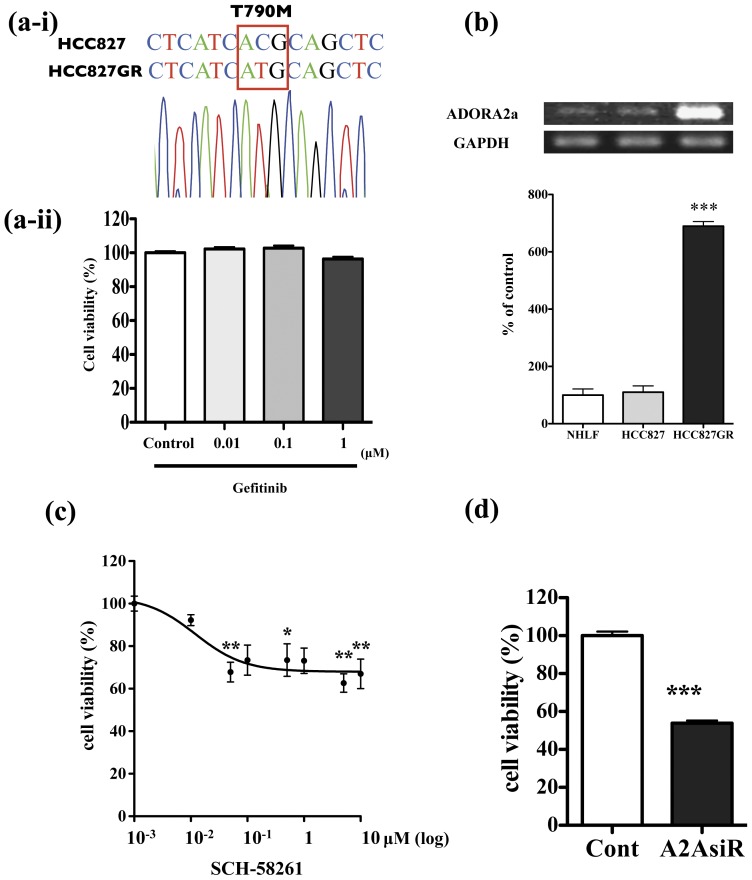
Effect of adenosine A2a receptors on the growth of gefitinib-resistant HCC827GR cells. (a-i) Sequence analysis in HCC827GR cell. A single nucleotide change C>T (EGFR exon 20) in DNA, which creates a missense change in the protein sequence which replaces a threonine with a methionine residue. (a-ii) HCC827GR cells were incubated with gefitinib for 2 days, and then cell viability was measured. (b) Upper: Representative RT-PCR for mRNAs of adenosine A2a receptor (ADORA2a) and GAPDH, an internal standard, in each cell type. Lower: The intensity of the bands was determined semi-quantitatively using ImageJ. The values for ADORA2a mRNA were normalized by the value for GAPDH mRNA. Data represent the mean with S.E.M. of 3 independent samples (***p<0.001 vs. NHLF). (c) HCC827GR cells were incubated for 7 days with the selective adenosine A2a receptor antagonist SCH-58261 (10 nM–10 µM), and then cell viability was measured (*p<0.05, **p<0.01 vs. non-treated group). (d) Treatment with siRNA targeting adenosine A2a receptors to HCC827GR cells for 3 days significantly decreased cell viability of HCC827GR cells (***p<0.001 vs. non-treated group).

To confirm the function of adenosine A2a receptors on gefiitnib-resistance to NSCLC, we generated anaother gefitinib-resistant HCC827GR cells by exposing HCC827 cells to increasing concentrations of gefitinib for 1.5 month. According to a sequence analysis, HCC827GR cells had second point mutation, due to caused mainly drug resistance, 2369 C>T in EGFR exon 20–21, which led to the transitions Thr790Met (**Figure 7a-i**). Therefore, the addition of gefitinib (0.01 µM–1 µM) for 2 days did not affect the growth of HCC827GR cells (**Figure 7a-ii**). The expression of adenosine A2a receptor mRNA was dramatically increased in HCC827GR cells compared to HCC827 and NHLF cells (**Figure 7b**, p<0.001 vs. NHLF). Under these conditions, the selective adenosine A2a receptor antagonist SCH-58261 (10 nM–1 µM) for 7 days produced a concentration-dependent decrease in HCC827GR cell growth (**Figure 7c**, p<0.05, p<0.001 vs. non-treated group). Furthermore, treatment with siRNA targeting adenosine A2a receptors also produced a significant decrease in HCC827GR cell growth (**Figure 7d**: p<0.001 vs. non-treated group).

## Discussion

GPCRs have traditionally been associated with many of the functions of differentiated, post-mitotic cells. On the other hand, GPCRs are also expressed in proliferating cells and contribute to embryogenesis, tissue remodeling and repair, inflammation, angiogenesis, normal cell growth and cancer.

Among them, protease-activated receptors (PARs), chemokine receptors and receptors for bio-active lipids such as LPA and sphingosine-1-phosphate (S1P) have been implicated in aberrant cell proliferation in a wide variety of cancer cells. Furthermore, neuropeptides such as endothelin, bradykinin, neuromedin B, cholecystokinin and angiotensin II activate their cognate GPCRs to stimulate cell proliferation in various cell types, and play a crucial role in many aggressive human cancers, including small-cell lung cancer (SCLC), pancreatic cancer, head and neck squamous cell carcinoma (HNSCC), and prostate cancer [18], [19]. However, there have been few reports about the critical role of GPCRs in gefitinib-resistant NSCLC cells, such as H1975 cells. A better understanding of the role of GPCRs in NSCLC can come from multiple analyses for gene-expression profiling. Microarray studies are likely to be useful tools for this purpose. Therefore, we performed a GPCR microarray analysis for gefitinib-resistant H1975 cells compared to normal human lung fibroblast cells. By profiling the expression of mRNAs encoding GPCRs, we found a large number of GPCRs overexpressed in H1975 cells. It has been recognized that many GPCRs that were overexpressed in various cancer types promote the growth of tumor cells when activated by circulating or locally produced ligands. Among GPCRs, GRP87 was listed as the most up-regulated GPCR in the present GPCR array. After this array, we validated the up-regulated expression of GRP87 mRNA and its protein using RT-PCR and western blotting, compared to those observed in NHLF cells. In H1975 cells, Gs-, Gi- and Gq-coupled GPCRs were overexpressed to almost the same extent, whereas some Gi-coupled GPCRs were down-regulated in H1975 cells.

The activation of A2a receptors has been reported to lead to immunosuppressive effects, which decreases anti-tumoral immunity and thus encourages tumor growth. In behavioral studies, it has been suggested that A2a antagonists should be added to immunotherapeutic protocols for cancer to enhance tumor immunotherapy. These compounds have already been shown to be safe in trials with A2a antagonists in treatment of Parkinson's disease [20]. In the present study, we investigated whether the specific agonists/antagonist which act on GPCRs that were up-regulated or down-regulated in H1975 cells could affect the growth of H1975 cells. Among the GPCRs listed, a selective antagonist of adenosine A2a receptor, which was one of the commercially available ligands for GPCRs overexpressed in H1975 cells, produced a dose-dependent decrease in H1975 cell viability. Similar to the antagonist, treatment with siRNA targeting adenosine A2a receptors produced a significant decrease in H1975 cell viability. To further investigate the role of adenosine A2a receptors in gefiitnib-resistance to NSCLC, we generated another gefitinib-resistant clone by exposing gefitinib to gefitinib-sensitive NSCLC cells, HCC827 cells. Like H1975 cells, the expression of adenosine A2a receptor mRNA was dramatically increased in geftinib-resistant HCC827GR cells compared to HCC827 and NHLF cells. Under these conditions, treatment with either the selective adenosine A2a receptor antagonist or siRNA targeting adenosine A2a receptors produced a significant decrease in HCC827GR cell growth. These findings suggest that, although further *in vivo* studies are still needed, the ability to interfere with adenosine A2a receptors may provide unique opportunities for the prevention and treatment of NSCLC. On the other hand, selective agonists for adenosine A2a receptor, angiotensin II receptor-like 1, purinergic receptor P2Y G-protein coupled 2, prostaglandin I_2_ receptor (IP), 5-hydroxytryptamine receptor 2B and arginine vasopressin receptor 1A had no effect on H1975 cell viability, which reflects the complex functions of GPCRs expressed in these cells. Many of the ligands or inhibitors for GPCRs that were up- or down-regulated in H1975 cells were not available for the present study. For instance, it is considered that GPR87, which was the largest overexpressed GPCR in H1975 cells, plays a crucial role in the p53-dependent survival of cancer cells exposed to DNA damage [21]. However, the specific ligand and its antagonist have not yet been identified. Since there is no doubt that GPCRs may be key players in the regulation of various pathophysiological responses, including cancer development and progression, it is likely that approaches involving interference with RNA will allow us to better understand the specialized functions of GPCRs expressed in gefitinib-resistant NSCLC cells.

In conclusion, we demonstrated that a large number of GPCRs were up-regulated while some were down-regulated via functional crosstalk between EGFR downstream and GPCR transcription in the development of gefitinib-resistance in NSCLC.

## Supporting Information

Figure S1 **List of GPCR gene symbols in microarray.** TaqMan Array Human GPCR card which allows us to quantify the expression of mRNAs that encode GPCRs from 50 subfamilies (343 receptors, not including the odorant, olfactory, gustatory and pheromone receptors), was used.
(PDF)

Figure S2 **Effects of several GPCR agonists on the growth of H1975 cells.** H1975 cells were incubated for 2 days with each GPCR ligand (1, 10 µM) and MTT assay was then performed. The cell viability data represents the mean with S.E.M. of 5 independent samples.
(PDF)
