# Correction: Multiple Analyses of G-Protein Coupled Receptor (GPCR) Expression in the Development of Gefitinib-Resistance in Transforming Non-Small-Cell Lung Cancer

**DOI:** 10.1371/annotation/49257f53-8cb1-431b-be64-7b410598b845

**Published:** 2013-08-08

**Authors:** Naoko Kuzumaki, Atsuo Suzuki, Michiko Narita, Takahiro Hosoya, Atsumi Nagasawa, Satoshi Imai, Kohei Yamamizu, Hiroshi Morita, Tsutomu Suzuki, Yohei Okada, Hirotaka James Okano, Jun K. Yamashita, Hideyuki Okano, Minoru Narita

The correct e-mail address for corresponding author Naoko Kuzumaki (NK) is kuzumaki@z8.keio.jp.

Figure legends 4 and 5 appear in reverse order. The following are the correct legends:

Figure 4. Expression ratio of Gα subunit (Gs, Gi and Gq) in H1975 cells. The percentage of expression pattern of Gα subunit (Gs: red, Gi: blue and Gq: green) in the up- or down-regulated GPCRs of H1975 cells compared to NHLF is shown.

Figure 5. High levels of GPR87 expression in H1975 cells. (a) Upper: Representative RT-PCR for mRNAs of GPR87 and GAPDH, an internal standard, in each cell type. Lower: The intensity of the bands was determined semi-quantitatively using ImageJ. The values for GPR87 mRNA were normalized by the value for GAPDH mRNA. Data represent the mean with S.E.M. of 3 independent samples (***p<0.001 vs. NHLF). (b) Upper: Representative Western blots of GPR87 Lower: Representative Western blots of GPR87 in membranous fractions of H1975 cells. Each column represents the mean with S.E.M. of 3 independent samples (**p<0.001 vs. non-treated group).

The sub-heading of the Results section, "Changes in the expression ratios of Ga subunits (Gs, Gi and Gq) in H1975 cells", contains two erroneous citations. The second and third paragraphs should read:

 The mRNA and its protein expressions of GRP87, which was the most up-regulated GPCR found in the GPCR array, were significantly up-regulated in H1975 cells compared to those in NHLF cells (Figure 5a: p<0.001 vs. NHLF, Figure 5b: p< 0.01 vs. NHLF).

 Among up-regulated GPCRs in H1975 cells, Gs-, Gi- and Gq-coupled GPCRs were expressed almost equally. Among downregulated GPCRs, Gi-coupled GPCRs were dominantly expressed in H1975 cells (Figure 4). 

