# Supplementary material for: Multiple Analyses of G-Protein Coupled Receptor (GPCR) Expression in the Development of Gefitinib-Resistance in Transforming Non-Small-Cell Lung Cancer
Source: PLoS One. 2012 Oct 29;7(10):e44368. doi: 10.1371/journal.pone.0044368 (PMC3483178; doi:10.1371/journal.pone.0044368)
Supplement: Figure S1 — List of GPCR gene symbols in microarray. TaqMan Array Human GPCR card which allows us to quantify the expression of mRNAs that encode GPCRs from 50 subfamilies (343 receptors, not including the odorant, olfactory, gustatory and pheromone receptors), was used. (PDF) [file pone.0044368.s001.pdf]

# Figure S1 List of 384 GPCR gene symbols in microarray

| Symbol | Description | Symbol | Description |
|---|---|---|---|
| GABBR1 | gamma-aminobutyric acid (GABA) B receptor, 1 | GPR113 | G protein-coupled receptor 113 |
| MRGPRX4 | MAS-related GPR, member X4 | PTGER1 | prostaglandin E receptor 1 |
| PTHR1 | parathyroid hormone receptor 1 | MRGPRX3 | MAS-related GPR, member X3 |
| FZD10 | frizzled homolog 10 (Drosophila) | OR2C3 | olfactory receptor, family 2, subfamily C, member 3 |
| BDKRB2 | bradykinin receptor B2 | GPR6 | G protein-coupled receptor 6 |
| P2RY10 | purinergic receptor P2Y, G-protein coupled, 10 | RLN3R1 | relaxin family peptide receptor 3 |
| GPR12 | G-protein coupled receptor 12 | TAAR8 | trace amine associated receptor 8 |
| TAAR2 | trace amine-associated receptor 2 | GPR150 | G protein-coupled receptor 150 |
| CHRM1 | cholinergic receptor, muscarinic 1 | TAAR1 | trace amine-associated receptor 1 |
| C11ORF4 | mitochondrial ribosomal protein L49 | GRM2 | glutamate receptor, metabotropic 2 |
| OPN3 | opsin 3 | FPRL2 | formyl peptide receptor 3 |
| GCGR | glucagon receptor | GPR83 | G protein-coupled receptor 83 |
| GRPR | gastrin-releasing peptide receptor | GPR25 | G protein-coupled receptor 25 |
| RARS | arginyl-tRNA synthetase | GPR62 | G protein-coupled receptor 62 |
| ADRA2B | adrenergic, alpha-2B-, receptor | HRH2 | histamine receptor H2 |
| CMKLR1 | chemokine-like receptor 1 | GPR4 | G protein-coupled receptor 4 |
| XCR1 | chemokine (C motif) receptor 1 | GPR40 | free fatty acid receptor 1 |
| EMR1 | egf-like module containing mucin-like hormone receptor-like 1 | MELL1 | mel transforming oncogene-like 1 |
| MC2R | melanocortin 2 receptor (adrenocorticotropic hormone) | CHRM5 | cholinergic receptor, muscarinic 5 |
| MC3R | melanocortin 3 receptor | OXER1 | oxoeicosanoid (OXE) receptor 1 |
| GPR133 | G protein-coupled receptor 133 | GNRHR | gonadotropin-releasing hormone receptor |
| DRD1 | dopamine receptor D1 | GPR120 | G protein-coupled receptor 120 |
| CYSLTR2 | cysteinyl leukotriene receptor 2 | GPR111 | G protein-coupled receptor 111 |
| GPR142 | G protein-coupled receptor 142 | EDG6 | endothelial differentiation, lysophosphatidic acid G-protein-coupled receptor, 6 |
| GHSR | growth hormone secretagogue receptor | VN1R5 | vomeronasal 1 receptor 5 |
| HTR1B | 5-hydroxytryptamine (serotonin) receptor 1B | HTR1A | 5-hydroxytryptamine (serotonin) receptor 1A |
| BLR1 | chemokine (C-X-C motif) receptor 5 | MC4R | melanocortin 4 receptor |
| GPR | P2 phage tail completion protein R | TAAR5 | trace amine-associated receptor 5 |
| BAI2 | brain-specific angiogenesis inhibitor 2 | GPR32 | G protein-coupled receptor 32 |
| GPR1 | G protein-coupled receptor 1 | PTGER4 | prostaglandin E receptor 4 (subtype EP4) |
| GPR65 | G-protein coupled receptor 65 | GPR135 | G protein-coupled receptor 135 |
| GPR45 | G protein-coupled receptor 45 | ADORA3 | adenosine A3 receptor |
| P2RY13 | purinergic receptor P2Y, G-protein coupled, 13 | ADORA1 | adenosine A1 receptor |
| FZD2 | frizzled homolog 2 (Drosophila) | SSTR3 | somatostatin receptor 3 |
| GPR141 | G protein-coupled receptor 141 | HTR7 | 5-hydroxytryptamine (serotonin) receptor 7 |
| GPR174 | G protein-coupled receptor 174 | OR7C2 | olfactory receptor, family 7, subfamily C, member 2 |
| GPR23 | G protein-coupled receptor 23 | TAAR6 | trace amine associated receptor 6 |
| GPR24 | melanin-concentrating hormone receptor 1 | CCRL2 | chemokine (C-C motif) receptor-like 2 |
| ACTB | actin, beta | FY | fuzzy |
| CYSLTR1 | cysteinyl leukotriene receptor 1 | FKSG83 | FKSG83 |
| GPR63 | G protein-coupled receptor 63 | TAAR9 | trace amine associated receptor 9 |
| TACR2 | tachykinin receptor 2 | MRGPRX1 | MAS-related GPR, member X1 |
| CCR3 | chemokine (C-C motif) receptor 3 | PPIA | peptidylprolyl isomerase A |
| GPR147 | neuropeptide FF receptor 1 | AGTRL1 | angiotensin II receptor-like 1 |
| TBXA2R | thromboxane A2 receptor | FZD9 | frizzled homolog 9 (Drosophila) |
| GPR21 | G protein-coupled receptor 21 | GPR31 | G protein-coupled receptor 31 |
| GPR126 | G protein-coupled receptor 126 | P2RY8 | purinergic receptor P2Y, G-protein coupled, 8 |
| SSTR5 | somatostatin receptor 5 | OPRL1 | opiate receptor-like 1 |
| HTR1F | 5-hydroxytryptamine (serotonin) receptor 1F | POLR2A | polymerase (RNA) II (DNA directed) polypeptide A |
| BAI1 | brain-specific angiogenesis inhibitor 1 | CD97 | CD97 molecule |
| CCBP2 | chemokine binding protein 2 | VN1R2 | vomeronasal 1 receptor 2 |
| LONPL | lon peptidase 2, peroxisomal | CHRM3 | cholinergic receptor, muscarinic 3, cardiac |
| GPR68 | G protein-coupled receptor 68 | MAS1L | MAS1 oncogene-like |
| GPR148 | G protein-coupled receptor 148 | GIPR | gastric inhibitory polypeptide receptor |
| LANCL1 | LanC lantibiotic synthetase component C-like 1 (bacterial) | GPR30 | G protein-coupled estrogen receptor 1 |
| NPY1R | neuropeptide Y receptor Y1 | PPYR1 | pancreatic polypeptide receptor 1 |
| C3AR1 | complement component 3a receptor 1 | CALCR | calcitonin receptor |
| OXGR1 | oxoglutarate (alpha-ketoglutarate) receptor 1 | SLC26A7 | solute carrier family 26, member 7 |
| HTR1E | 5-hydroxytryptamine (serotonin) receptor 1E | GPR152 | G protein-coupled receptor 152 |
| GPR51 | gamma-aminobutyric acid (GABA) B receptor, 2 | CCR4 | chemokine (C-C motif) receptor 4 |
| GPR8 | neuropeptides B/W receptor 2 | GPR34 | G protein-coupled receptor 34 |
| GPR173 | G protein-coupled receptor 173 | GPR77 | G protein-coupled receptor 77 |
| GPR101 | G protein-coupled receptor 101 | SSTR2 | somatostatin receptor 2 |
| HCRTR2 | hypocretin (orexin) receptor 2 | DRD5 | dopamine receptor D5 |

| Symbol | Description | Symbol | Description |
|---|---|---|---|
| MATK | megakaryocyte-associated tyrosine kinase | MLNR | motilin receptor |
| EDG8 | sphingosine-1-phosphate receptor 5 | MRGPRD | MAS-related GPR, member D |
| GPR146 | G protein-coupled receptor 146 | MTNR1B | melatonin receptor 1B |
| LPHN1 | latrophilin 1 | NMBR | neuromedin B receptor |
| MAS1 | MAS1 oncogene | LGR5 | eucine rich repeat containing G protein coupled receptor 5 |
| ADRA1B | adrenergic, alpha-1B-, receptor | LHCGR | uteinizing hormone/choriogonadotropin receptor |
| GPR85 | G protein-coupled receptor 85 | LPHN3 | atrophilin 3 |
| GPRC5B | G protein-coupled receptor, family C, group 5, member B | MC5R | melanocortin 5 receptor |
| GPR54 | G protein-coupled receptor 54 | HTR5A | 5-hydroxytryptamine (serotonin) receptor 5A |
| OXTR | oxytocin receptor | HTR6 | 5-hydroxytryptamine (serotonin) receptor 6 |
| OR2A4 | OR2A4 olfactory receptor, family 2, subfamily A, member 4 | IL8RA | nterleukin 8 receptor, alpha |
| LPHN2 | latrophilin 2 | IL8RB | interleukin 8 receptor, beta |
| PTGER2 | prostaglandin E receptor 2 (subtype EP2), 53kDa | HRH4 | histamine receptor H4 |
| P2RY5 | lysophosphatidic acid receptor 6 | HTR2A | 5-hydroxytryptamine (serotonin) receptor 2A |
| LGR7 | relaxin/insulin-like family peptide receptor 1 | HTR2C | 5-hydroxytryptamine (serotonin) receptor 2C |
| PTGFR | prostaglandin F receptor (FP) | HTR4 | 5-hydroxytryptamine (serotonin) receptor 4 |
| FZD6 | frizzled homolog 6 (Drosophila) | GRM7 | glutamate receptor, metabotropic 7 |
| GRM4 | glutamate receptor, metabotropic 4 | GRM8 | glutamate receptor, metabotropic 8 |
| CALCRL | calcitonin receptor-like | HCRTR1 | hypocretin (orexin) receptor 1 |
| EBI2 | Epstein-Barr virus induced gene 2 (lymphocyte-specific G protein-coupled receptor) | HRH3 | histamine receptor H3 |
| CELSR2 | adherin, EGF LAG seven-pass G-type receptor 2 (flamingo homolog, Drosophila) | GRM1 | glutamate receptor, metabotropic 1 |
| EDG5 | sphingosine-1-phosphate receptor 2 | GRM3 | glutamate receptor, metabotropic 3 |
| GPR37 | G protein-coupled receptor 37 (endothelin receptor type B-like) | GRM5 | glutamate receptor, metabotropic 5 |
| GPR52 | G protein-coupled receptor 52 | GRM6 | glutamate receptor, metabotropic 6 |
| CCR5 | chemokine (C-C motif) receptor 5 | GPR97 | G protein-coupled receptor 97 |
| GCNT2 | glucosaminyl (N-acetyl) transferase 2, I-branching enzyme (I blood group) | GPRC5C | G protein-coupled receptor, family C, group 5, member C |
| GPR88 | G-protein coupled receptor 88 | GPRC5D | G protein-coupled receptor, family C, group 5, member D |
| EDG2 | lysophosphatidic acid receptor 1 | GPRC6A | G protein-coupled receptor, family C, group 6, member A |
| ADMR | G protein-coupled receptor 182 | GPR73L1 | prokineticin receptor 2 |
| GPR44 | G protein-coupled receptor 44 | GPR74 | G protein-coupled receptor 74 |
| MRGPRF | MAS-related GPR, member F | GPR78 | G protein-coupled receptor 78 |
| ADRA1D | adrenergic, alpha-1D-, receptor | GPR84 | G protein-coupled receptor 78 |
| SMO | smoothened homolog (Drosophila) | GPR27 | G protein-coupled receptor 27 |
| PTGIR | prostaglandin I2 (prostacyclin) receptor (IP) | GPR37L1 | G protein-coupled receptor 37 like 1 |
| ELTD1 | EGF, latrophilin and seven transmembrane domain containing 1 | GPR50 | G protein-coupled receptor 50 |
| HTR2B | 5-hydroxytryptamine (serotonin) receptor 2B | GPR73 | prokineticin receptor 1 |
| NMUR1 | neuromedin U receptor 1 | GPR17 | G protein-coupled receptor 17 |
| NTSR1 | neurotensin receptor 1 (high affinity) | GPR18 | G protein-coupled receptor 18 |
| C5R1 | complement component 5a receptor 1 | GPR20 | G protein-coupled receptor 20 |
| P2RY12 | purinergic receptor P2Y, G-protein coupled, 12 | GPR26 | G protein-coupled receptor 26 |
| CCR10 | chemokine (C-C motif) receptor 10 | GPR114 | G protein-coupled receptor 114 |
| TBP | TATA binding protein | GPR123 | G protein-coupled receptor 123 |
| 18S | | GPR128 | G protein-coupled receptor 128 |
| CHRM2 | cholinergic receptor, muscarinic 2 | GPR145 | melanin-concentrating hormone receptor 2 |
| FZD1 | frizzled homolog 1 (Drosophila) | GPBAR1 | G protein-coupled bile acid receptor 1 |
| AVPR2 | arginine vasopressin receptor 2 | GPR10 | prolactin releasing hormone receptor |
| VIPR2 | vasoactive intestinal peptide receptor 2 | GPR103 | G protein-coupled receptor 103 |
| GPR124 | G protein-coupled receptor 124 | GPR112 | G protein-coupled receptor 112 |
| F2R | coagulation factor II (thrombin) receptor | GALR3 | galanin receptor 3 |
| SSTR1 | somatostatin receptor 1 | GHRHR | rowth hormone releasing hormone receptor |
| B2M | beta-2 microglobulin | GLP1R | glucagon-like peptide 1 receptor |
| FPRL1 | formyl peptide receptor-like 1 | GLP2R | glucagon-like peptide 2 receptor |
| TACR3 | tachykinin receptor 3 | EMR3 | egf-like module containing, mucin-like, hormone receptor-like 3 |
| TAS1R1 | taste receptor, type 1, member 1 | F2RL3 | coagulation factor II (thrombin) receptor-like 3 |
| TRBV5-4 | T cell receptor beta variable 5-4 | FSHR | follicle stimulating hormone receptor |
| TRHR | thyrotropin-releasing hormone receptor | GALR1 | galanin receptor 1 |
| OPN1SW | opsin 1 (cone pigments), short-wave-sensitive | CASR | calcium-sensing receptor |
| OPN4 | opsin 4 | CCKAR | cholecystokinin A receptor |
| OPRD1 | opioid receptor, delta 1 | CCKBR | cholecystokinin B receptor |
| OPRK1 | opioid receptor, kappa 1 | CCR2 | chemokine (C-C motif) receptor 2 |
| NMUR2 | neuromedin U receptor 2 | AGTR1 | angiotensin II receptor, type 1 |
| NPY2R | neuropeptide Y receptor Y2 | AGTR2 | angiotensin II receptor, type 2 |
| NPY5R | neuropeptide Y receptor Y5 | BAI3 | brain-specific angiogenesis inhibitor 3 |
| NTSR2 | neurotensin receptor 2 | BRS3 | bombesin-like receptor 3 |

**Part 2**

| Symbol | Description |
|---|---|
| TSHR | thyroid stimulating hormone receptor |
| UTS2R | urotensin 2 receptor |
| ADCYAP1 | adenylate cyclase activating polypeptide 1 |
| ADRA1A | adrenergic, alpha-1A-, receptor |
| ADRA2A | adrenergic, alpha-2A-, receptor |
| ADRB3 | adrenergic receptor, beta 3 |
| CCR6 | chemokine (C-C motif) receptor 6 |
| CCR7 | chemokine (C-C motif) receptor 7 |
| CCR8 | hemokine (C-C motif) receptor 8 |
| CCR9 | chemokine (C-C motif) receptor 9 |
| CNR1 | cannabinoid receptor 1 |
| CNR2 | cannabinoid receptor 2 |
| CRHR1 | corticotropin releasing hormone receptor 1 |
| CRHR2 | corticotropin releasing hormone receptor 2 |
| CX3CR1 | chemokine (C-X3-C motif) receptor 1 |
| CXCR3 | chemokine (C-X-C motif) receptor 3 |
| CXCR4 | chemokine (C-X-C motif) receptor 4 |
| CXCR6 | chemokine (C-X-C motif) receptor 6 |
| DRD2 | dopamine receptor D2 |
| DRD3 | dopamine receptor D3 |
| DRD4 | dopamine receptor D4 |
| EDNRB | endothelin receptor type B |
| SCTR | secretin receptor |
| SSTR4 | somatostatin receptor 4 |
| SUCNR1 | succinate receptor 1 |
| TACR1 | tachykinin receptor 1 |
| PTPN22 | protein tyrosine phosphatase, non-receptor type 22 (lymphoid) |
| RGR | retinal G protein coupled receptor |
| RHO | rhomboid |
| RRH | retinal pigment epithelium derived rhodopsin homolog |
| OPRM1 | opioid receptor, mu 1 |
| OR7E5P | olfactory receptor, family 7, subfamily E, member 5 pseudogene |
| P2RY14 | purinergic receptor P2Y, G-protein coupled, 14 |
| P2RY6 | pyrimidinergic receptor P2Y, G-protein coupled, 6 |
| PCDH15 | protocadherin-related 15 |
| PTGDR | prostaglandin D2 receptor (DP) |
| PTGER3 | prostaglandin E receptor 3 (subtype EP3) |
| PTHR2 | parathyroid hormone 2 receptor |
| F2RL2 | coagulation factor II (thrombin) receptor-like 2 |
| CCRL1 | chemokine (C-C motif) receptor-like 1 |
| P2RY1 | purinergic receptor P2Y, G-protein coupled, 1 |
| EDG3 | sphingosine-1-phosphate receptor 3 |
| EDG1 | sphingosine-1-phosphate receptor 1 |
| GPR61 | G protein-coupled receptor 61 |
| GNRHR2 | gonadotropin-releasing hormone (type 2) receptor 2 |
| BDKRB1 | bradykinin receptor B1 |
| FZD4 | frizzled homolog 4 (Drosophila) |
| MRGPRE | MAS-related GPR, member E |
| AVPR1B | arginine vasopressin receptor 1B |
| PR153 | GPR153-Hs00664129_s1 |
| GPR161 | G protein-coupled receptor 161 |
| CCR1 | chemokine (C-C motif) receptor 1 |
| VN1R1 | vomeronasal 1 receptor 1 |
| FZD7 | frizzled homolog 7 (Drosophila) |
| FZD8 | frizzled homolog 8 (Drosophila) |
| GPR19 | G protein-coupled receptor 19 |
| GPR110 | G protein-coupled receptor 110 |
| GPR81 | G protein-coupled receptor 81 |
| ADORA2B | adenosine A2b receptor |
| GPR43 | free fatty acid receptor 2 |
| GALR2 | galanin receptor 2 |
| GPR39 | G protein-coupled receptor 39 |
| PHGDH | phosphoglycerate dehydrogenase |
| FZD5 | frizzled homolog 5 (Drosophila) |
| GPR115 | G protein-coupled receptor 115 |
| VIPR1 | vasoactive intestinal peptide receptor 1 |
| GPR143 | G protein-coupled receptor 143 |
| GPR92 | G protein-coupled receptor 92 |
| LGR6 | leucine-rich repeat-containing G protein-coupled receptor 6 |
| HRH1 | histamine receptor H1 |
| LANCL2 | LanC lantibiotic synthetase component C-like 2 |
| PTAFR | platelet-activating factor receptor |
| GPR56 | G protein-coupled receptor 56 |
| RPLP0 | acidic ribosomal protein P0 |
| GPR15 | G protein-coupled receptor 15 |
| GPR87 | G protein-coupled receptor 87 |
| LTB4R | leukotriene B4 receptor |
| CELSR1 | cadherin, EGF LAG seven-pass G-type receptor 1 (flamingo homolog, Drosophila) |
| GPRC5A | G protein-coupled receptor, family C, group 5, member A |
| FZD3 | frizzled homolog 3 (Drosophila) |
| P2RY2 | purinergic receptor P2Y, G-protein coupled, 2 |
| HDAC3 | Histone deacetylase 3 |
| GPR7 | G protein-coupled receptor 7 |
| GPR125 | G protein-coupled receptor 125 |
| GPR35 | G protein-coupled receptor 35 |
| ADRB1 | adrenergic receptor, beta 1 |
| GPR82 | G protein-coupled receptor 82 |
| PGK1 | phosphoglycerate kinase 1 |
| HTR1D | 5-hydroxytryptamine (serotonin) receptor 1D |
| F2RL1 | coagulation factor II (thrombin) receptor-like 1 |
| P2RY11 | purinergic receptor P2Y, G-protein coupled, 11 |
| P2RY4 | pyrimidinergic receptor P2Y, G-protein coupled, 4 |
| SENP3 | SUMO1/sentrin/SMT3 specific peptidase 3 |
| LTB4R2 | leukotriene B4 receptor 2 |
| CELSR3 | cadherin, EGF LAG seven-pass G-type receptor 3 |
| EDG4 | lysophosphatidic acid receptor 2 |
| GPR22 | G protein-coupled receptor 22 |
| HPRT1 | hypoxanthine phosphoribosyltransferase 1 |
| MRGPRX2 | MAS-related GPR, member X2 |
| IPO8 | importin 8 |
| ADRB2 | adrenergic, beta-2-, receptor, surface |
| HMBS | hydroxymethylbilane synthase |
| LGR4 | leucine-rich repeat-containing G protein-coupled receptor 4 |
| FPR1 | formyl peptide receptor 1 |
| GPR3 | G-protein coupled receptor 3 |
| EMR2 | egf-like module containing, mucin-like, hormone receptor-like 2 |
| CHRM4 | cholinergic receptor, muscarinic 4 |
| GPR75 | G protein-coupled receptor 75 |
| NPY6R | neuropeptide Y receptor Y6 |
| GUSB | glucuronidase, beta |
| ADORA2A | adenosine A2a receptor |
| TFRC | transferrin receptor (p90, CD71) |
| GPR17 | G protein-coupled receptor 17 |
| EDNRA | endothelin receptor type A |
| GPR116 | G protein-coupled receptor 116 |
| GPR55 | G protein-coupled receptor 55 |
| MC1R | melanocortin 1 receptor |
| GPR160 | G protein-coupled receptor 160 |
| EDG7 | endothelial differentiation, lysophosphatidic acid G-protein-coupled receptor, 7 |
| MASS1 | monogenic, audiogenic seizure susceptibility 1 |
| GPR64 | G protein-coupled receptor 64 |
| MTNR1A | melatonin receptor 1A |
| AVPR1A | arginine vasopressin receptor 1A |
| TM7SF1 | G protein-coupled receptor 137B |

**Part 3**
