# Supplementary material for: Multiple Analyses of G-Protein Coupled Receptor (GPCR) Expression in the Development of Gefitinib-Resistance in Transforming Non-Small-Cell Lung Cancer
Source: PLoS One. 2012 Oct 29;7(10):e44368. doi: 10.1371/journal.pone.0044368 (PMC3483178; doi:10.1371/journal.pone.0044368)
Supplement: Figure S2 — Effects of several GPCR agonists on the growth of H1975 cells. H1975 cells were incubated for 2 days with each GPCR ligand (1, 10 µM) and MTT assay was then performed. The cell viability data represents the mean with S.E.M. of 5 independent samples. (PDF) [file pone.0044368.s002.pdf]

Figure S2 Effects of several GPCR agonists on the growth of H1975 cells

Ligand name (targeted receptor)	Dose	Cell viability (% of control)
CGS-21680 *(Adenosine A2a receptor)*	1 μM	93.86 ±1.40
	10 μM	102.8 ±1.28
Angiotensin II *(Angiotensin II receptor-like 1)*	1 μM	99.94 ±2.16
	10 μM	99.12 ±1.99
2-(Methylthio) adenosine 5'-triphosphate tetrasodium salt hydrate *(Purinergic receptor P2Y, G-protein coupled, 2)*	1 μM	99.81 ±2.36
	10 μM	102.3 ±1.87
Beraprost sodium *(Prostaglandin I_2 receptor (IP))*	1 μM	101. 3±0.89
	10 μM	98.94 ±2.27
BW723C86 *(5-hydroxytryptamine receptor 2B)*	1 μM	89.0 ±1.38
	10 μM	93.85 ±2.78
[Arg^8]-vasopressin acetate salt *(Arginine vasopressin receptor 1A)*	1 μM	101.0 ±1.14
	10 μM	98.22 ±2.66
